# Impact of scholarly activities on early postgraduate medical trainees: a cross-sectional study

**DOI:** 10.1186/s12909-026-09272-x

**Published:** 2026-05-01

**Authors:** Hidehiro Someko, Yuki Kataoka, Atsushi Mizuno, Yuji Nishizaki, Taro Shimizu, Yasuharu Tokuda, Toshiaki Shiojiri

**Affiliations:** 1 Department of Internal Medicine, Nagoya Tokushukai General Hospital, Kouzoujichoukita, 2-52, 487-0016 Kasugai, Japan; 2 Department of Healthcare Epidemiology, Graduate School of Medicine, Kyoto University, Yoshida Konoe-cho, Sakyo-ku, Room 225, Medical Faculty G Building, Kyoto, 606-8501 Japan; 3 Scientific Research WorkS Peer Support Group (SRWS-PSG), Osaka, Japan; 4 Center for Postgraduate Clinical Training and Career Development, Nagoya University Hospital, 65, Tsurumai-cho, Showa‑ku, Nagoya, Japan; 5 Center for Medical Education, Graduate School of Medicine, Nagoya University, 65, Tsurumai-cho, Showa‑ku, Nagoya, Japan; 6 Department of Internal Medicine, Kyoto Min-iren Asukai Hospital, Tanaka Asukai-cho 89, Sakyo-ku, Kyoto, Japan; 7 Department of International and Community Oral Health, Tohoku University Graduate School of Dentistry, 4-1, Seiryo-machi, Aoba-ku, Sendai, Miyagi Japan; 8 Department of Cardiovascular Medicine, St. Luke’s International Hospital, Tokyo, Japan; 9 Division of Medical Education, Juntendo University School of Medicine, Tokyo, Japan; 10 Department of Diagnostic and Generalist Medicine, Dokkyo Medical University Hospital, Mibu, Tochigi Japan; 11 Muribushi Okinawa Clinical Training Center, Urasoe City, Okinawa Japan; 12 Tokyo Foundation for Policy Research, Roppongi, Minato-ku, Tokyo, Japan; 13 General Internal Medicine, Asahi General Hospital, Asahi, Japan

**Keywords:** Medical education, Resident education, Research requirements, Academic activity, Residency training

## Abstract

**Introduction:**

While scholarly activities are considered essential for medical advancement and improved patient outcomes, the educational value and potential impact on postgraduate medical trainees' well-being has not been well studied. We examined whether clinical research experience and scholarly activity requirements during early postgraduate medical training affect trainees' competency and well-being.

**Methods:**

We conducted a cross-sectional study of Japanese early postgraduate medical trainees (postgraduate year [PGY]1–2) recruited from hospitals participating in the General Medicine In-Training Examination (GM-ITE) in January 2024 and from teaching hospitals with scholarly activity requirements that did not participate in GM-ITE. Participants were classified into three groups based on scholarly activity experience: no scholarly activity, scholarly activity without clinical research, and scholarly activity with clinical research. The primary outcome was evidence-based medicine (EBM) competency assessed using the Japanese version of the Assessing Competency in EBM (ACE) tool. Secondary outcomes included GM-ITE scores, future scholarly activity intentions, depression symptoms, and well-being measures. Associations between scholarly activity experience and outcomes were examined using multiple regression analysis for continuous outcomes and logistic regression analysis for binary outcomes, adjusting for potential confounders; missing data were addressed using multiple imputation.

**Results:**

Among 1,152 participants (1,150 from GM-ITE-participating hospitals and 2 from non-participating hospitals), 656 (57.0%) reported engaging in scholarly activities during early postgraduate medical training, with 60 (9.1%) conducting clinical research. After adjusting for potential confounders, compared to trainees without scholarly activity, the difference in ACE tool scores was 0.22 points (95% confidence interval [CI]: 0.02 to 0.41) for those who engaged in scholarly activities without clinical research and 0.40 points (95% CI: -0.02 to 0.83) for those who conducted clinical research. Stratification by program requirements revealed differences of 0.34 points (95% CI: 0.09 to 0.58) for voluntary scholarly activities and 0.09 points (95% CI: -0.19 to 0.37) for required activities. Trainees who conducted clinical research or engaged in required scholarly activities showed higher odds of future academic interest (adjusted odds ratio [OR]: 2.68 [95% CI: 1.33–5.37] and 1.52 [1.02–2.25], respectively), while those who engaged in scholarly activities without clinical research or without requirements showed similar odds to the reference group (adjusted OR: 1.27 [0.97–1.68] and 1.24 [0.88–1.74], respectively) Well-being measures showed minimal differences across groups.

**Conclusion:**

Scholarly activities during early postgraduate medical training, whether required by programs or not, had little impact on trainees' competency and well-being. However, trainees who engaged in clinical research showed increased interest in future academic activities, suggesting the importance of establishing supportive environments for interested trainees.

**Supplementary Information:**

The online version contains supplementary material available at 10.1186/s12909-026-09272-x.

## Introduction

Scholarly activities play a vital role in postgraduate medical training by enhancing trainees’ overall clinical competency, including evidence-based medicine skills [1, 2]. These activities not only contribute to medical advancement but also improve outcomes for patients [3].

Previous studies have demonstrated that scholarly activity during residency correlates with positive outcomes, including increased future publication productivity (odds ratio 4.25; 95% CI, 1.50–12.06) and higher satisfaction with training programs (odds ratio 1.50; 95% CI, 1.10–2.10) [4, 5]. However, scholarly activities may impose additional time demands and stress on trainees, with surveys indicating that more than half of academic trainees report psychological distress and elevated rates of depression and anxiety compared to the general population [6]. This tension between educational benefit and trainee well-being has created uncertainty about the optimal implementation timing of scholarly activities in medical training. While the Accreditation Council for Graduate Medical Education (ACGME) requires scholarly activities during residency training (typically postgraduate year [PGY] 2–6) and several studies have shown increased academic productivity under such requirements [7, 8], two critical knowledge gaps persist. First, while studies examining residency years (PGY2-6) have shown mixed results regarding the impact of scholarly activities on clinical competency - from no negative effects to potential interference with clinical training - evidence during earlier training years (PGY1-2) remains limited [9, 10]. Second, while training programs increasingly incorporate scholarly activities in early postgraduate education [11], their effects on trainee well-being and educational outcomes lack systematic evaluation.

This nationwide cross-sectional study examined the association between scholarly activities and both clinical competency and well-being among postgraduate trainees in Japan. We analyzed data from junior residents (the Japanese term for PGY-1 and PGY-2) who completed the General Medicine In-Training Examination (GM-ITE) and accompanying survey as well as residents from teaching hospitals with scholarly activity requirements who did not participate in the GM-ITE. Our primary objectives were to quantify: (i) the relationship between scholarly activity engagement and EBM competency scores, and (ii) the impact of scholarly activity requirements on trainees’ well-being.

## Methods

### Study design and participants

We conducted a cross-sectional study among junior residents in Japan. Participants were recruited through two distinct pathways based on their GM-ITE (General Medicine In-Training Examination) participation status. The first group consisted of PGY-1 and PGY-2 residents who took the GM-ITE on January 17 in 2024. The second group comprised PGY-2 residents from teaching hospitals requiring scholarly activities in their training program but not participating in GM-ITE. Since clinical research experience is uncommon among junior residents at most GM-ITE-participating hospitals in Japan, recruiting from GM-ITE-participating hospitals alone would have yielded insufficient numbers of junior residents with clinical research experience to enable meaningful comparisons across the three exposure groups. We focused on PGY-2 residents in this group as they were more likely to have completed their required scholarly activities by this stage of training.

Prior to participation, all residents were provided with a research document explaining data anonymization procedures, the voluntary nature of participation, and plans for publication of study results. Participants who provided informed consent were enrolled in the study, with the right to withdraw from the study at any time.

Participants who provided logically inconsistent responses were excluded from the analysis in both groups. For instance, if a respondent selected both “clinical research” as a type of scholarly activity they had engaged in and “no scholarly activity experience,” their data were deemed invalid and removed. The flow of participant selection is shown in Figure.

**Figure:**
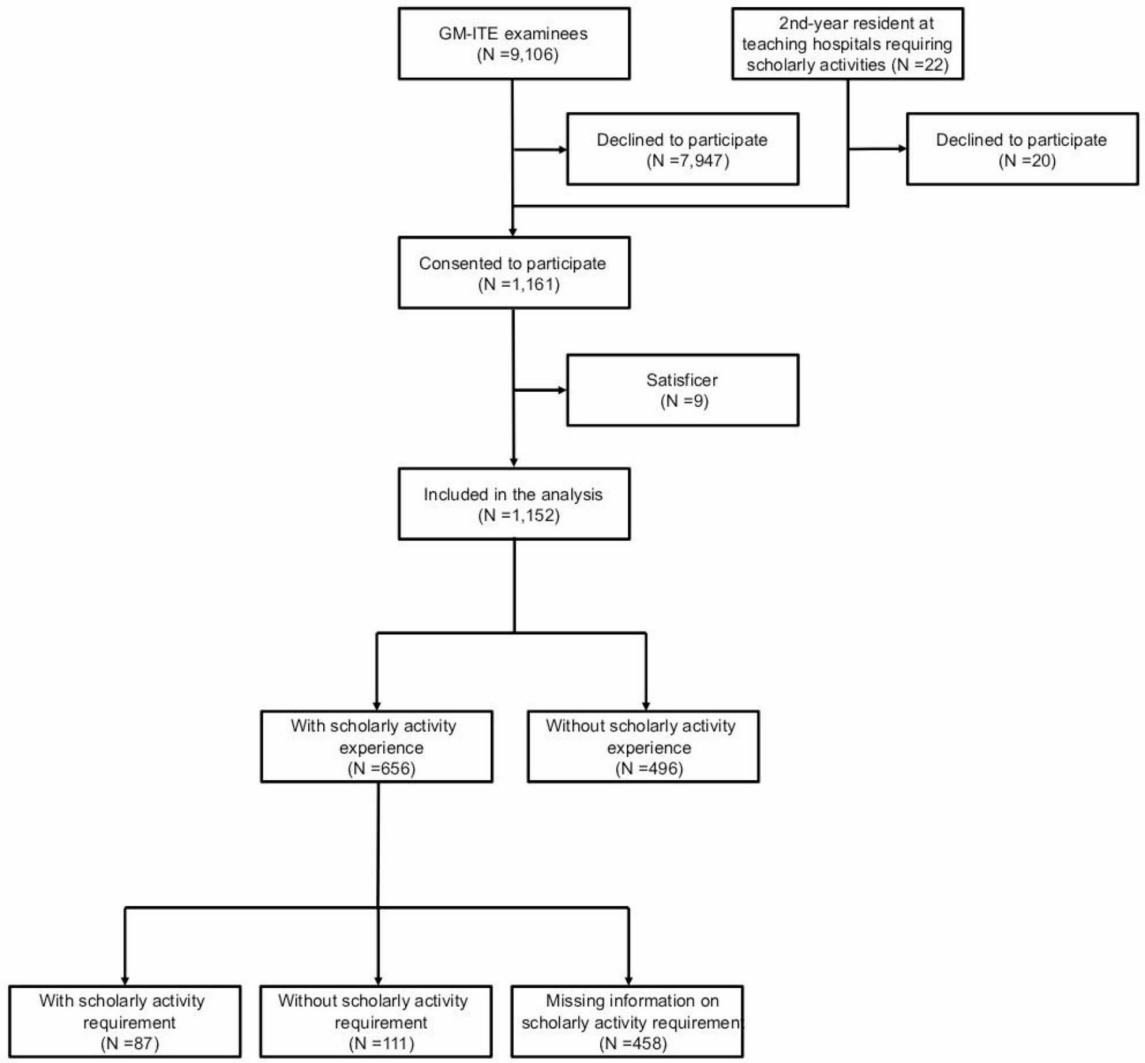
Participant flow chart. Abbreviation: GM-ITE General Medicine In-Training Examination

This study was approved by the Ethics Review Board of Japan Institute for Advancement of Medical Education Program (JAMEP); approval number 23 − 7. 

### General medicine in-training examination

The GM-ITE, modeled after the United States’ Internal Medicine In-Training Examination (IM-ITE), is a standardized assessment tool developed by the Japan Institute for Advancement of Medical Education Program (JAMEP) in 2011 [12]. The examination provides junior residents and training program directors with an objective assessment of clinical knowledge. It consists of 60 questions covering four main domains: medical interview and professionalism, symptomatology and clinical reasoning, physical examination and clinical procedures, and disease knowledge. Some questions incorporate multimedia elements including video and audio formats. The questions are developed annually by a committee of experienced physicians and undergo peer review by an independent committee. Only residents affiliated with authorized training hospitals are eligible to take the GM-ITE. 

### Data collection

Data collection occurred during different time periods for each group. For GM-ITE participants, data were collected from January 17 to March 31, 2024, immediately following their examination through the GM-ITE online platform. For non-GM-ITE participants, recruitment and data collection were conducted from March 1 to March 31, 2024.

We collected data through two separate web-based questionnaires. For GM-ITE participants, the survey was administered through the GM-ITE online platform. For non-GM-ITE participants from teaching hospitals requiring scholarly activities, data were collected via a web form. The form URL was first sent to hospital staff by the principal investigator, who then forwarded it to individual residents via email. This form included consent documentation, the study questionnaire, and the Japanese version of the Assessing Competency in EBM (ACE) tool [13], which participants completed through the online platform. 

### Definition of exposure and study groups

We defined scholarly activities as serving as the primary presenter or first author in any of the following formats: institutional presentations (case conferences or research meetings simulating academic presentations, excluding clinical pathology conferences and practice sessions for academic meetings), presentations at academic society meetings (national or regional), educational seminars organized by non-academic societies, publications in peer-reviewed academic journals, or publications in non-peer-reviewed commercial journals [1]. The content could include one or more of the following: case reports, clinical research, medical education research, basic research, or healthcare quality evaluations. Presentations and publications in any language were eligible. These definitions were also provided to participants in the survey instrument.

The content of scholarly activities was classified into six categories: case reports, which provide detailed documentation of unique clinical encounters and their learning points; clinical research, which investigates patient care approaches and their outcomes; medical education research, which examines and enhances teaching and learning methods in medicine; quality improvement research, which focuses on optimizing healthcare delivery systems and processes; basic research, which explores fundamental biological or physiological mechanisms; and letters to the editor. Based on this classification and program requirements, we examined two types of the exposures:

i) Content-based exposure: Residents were categorized into two groups - those who conducted scholarly activities with clinical research (including at least one clinical research activity) and those who conducted scholarly activities without clinical research (including at least one scholarly activity other than clinical research). We treated scholarly activities involving clinical research as a distinct exposure because, although EBM competency is not synonymous with research capability, participation in clinical research may provide more structured opportunities to practice core EBM processes (e.g., question formulation and critical appraisal) than other scholarly activities.
ii) Requirement-based exposure: Residents were categorized based on whether their scholarly activities were conducted as part of program requirements (required scholarly activities) or without such requirements (voluntary scholarly activities).

The comparison group for both exposures consisted of residents who did not engage in any scholarly activities during their training period. 

### Primary outcome

The primary outcome was EBM competency, assessed using the total score of the Japanese version of the ACE tool^9^. The ACE tool consists of a patient scenario, a clinical question, a search strategy, and a hypothetical article abstract, followed by 15 yes/no questions. It evaluates four of the five EBM steps: formulating a clinical question, searching the literature, critically appraising the evidence, and applying the evidence to the clinical scenario. The Japanese version was developed following the COSMIN (COnsensus-based Standards for the selection of health Measurement INstruments) guidelines [14] and has demonstrated acceptable test-retest reliability (ICC = 0.64). The tool has shown moderate correlation (*r* = 0.35) with the original English version of Fresno test, a validated assessment tool for EBM competency. The median completion time for the tool is approximately 14 min (847 s). All participants completed the ACE tool through an online platform. 

### Secondary outcomes

Secondary outcomes included GM-ITE scores, future scholarly activity intentions, depression symptoms, and well-being measures. Depression was assessed using the Japanese version of Patient Health Questionnaire-2 (PHQ-2) [15], a validated two-item screening tool that asks about loss of interest or pleasure and depressed mood during the past two weeks, with responses in a dichotomous yes/no format. Depression was considered present if participants answered “yes” to either of the two questions. The Japanese version of PHQ-2 has been validated and demonstrated a sensitivity of 76% and specificity of 87% for diagnosing major depression [16].

Six items from the Japanese version of Mini-Z 2.0 questionnaire [17] were used to assess well-being of junior residents: burnout, work satisfaction, work stress, work control, documentation time during work hours, and time spent on documentation after work. Burnout was considered present if respondents selected any of the top three options (scores 1–3) in the burnout question, ranging from “I felt completely burned out” to “I was beginning to burn out and had one or more symptoms of burnout.” Work satisfaction was defined as responses of “Agree” or “Strongly agree” (scores 4–5) to the statement “Overall, I am satisfied with my current job.” High work stress was defined as responses of “Strongly agree” or “Agree” (scores 1–2) to the statement “I have felt a great deal of stress because of my job.” Inadequate work control was defined as responses of “Poor” or “Marginal” (scores 1–2) to the question about control over workload. Insufficient documentation time during work hours was defined as responses of “Poor” or “Marginal” (scores 1–2) to the question about sufficiency of time for documentation. Excessive after-hours documentation was defined as responses of “Excessive” or “Moderately high” (scores 1–2) to the question about amount of time spent on electronic medical records at home. The Japanese version of Mini-Z 2.0’s single-item burnout measure has shown a sensitivity of 54% and specificity of 88% for burnout diagnosis [13].

Additionally, participants were asked about their intention to engage in scholarly activities after completing residency training. GM-ITE scores were collected for participants who took the examination. 

### Covariates

We collected data on hospital characteristics (university hospital vs. community hospital, urban vs. suburban location, with urban defined as government-designated cities, prefectural capitals, or cities with medical schools), work-related factors (weekly working hours, number of inpatient cases, monthly night shifts, self-study time), research-related factors (graduate school enrollment status, research experience before junior residency). Social support was assessed using nine items from the Brief Job Stress Questionnaire (BJSQ) [18]. The items evaluated support from superiors, co-workers, and spouse/family/friends in terms of ease of communication, reliability in times of trouble, and willingness to listen to personal matters, with each item rated on a 4-point scale. Information about scholarly activity requirements in the junior residency program was obtained through a survey of junior residency program directors.

As a proxy measure for institutional academic engagement, we collected data on the number of presentations per postgraduate trainee at conferences organized by the Japanese Society of Internal Medicine [19]. Although our study focused on junior residents, we included presentation data from both junior and senior residents (postgraduate years 3–6) because separate data for junior residents alone was not available in the society’s database. This combined metric was used as a potential confounding factor to account for the institutional academic atmosphere that might influence residents’ scholarly activities.

Basic demographic information included age, gender, and training year (PGY-1 or PGY-2), and location and type of training hospital, which were collected through the individual survey.

### Statistical analysis

Baseline characteristics were presented as numbers with percentages for categorical variables and means with standard deviations or medians with interquartile ranges for continuous variables, depending on their distribution. Multiple regression analysis was performed to examine group differences in continuous outcome variables (ACE tool scores and GM-ITE scores) while adjusting for potential confounders. For binary outcomes (PHQ-2 positive/negative status, individual items of 10-item Mini-Z questionnaire, and intention to engage in scholarly activities after residency), logistic regression analysis was conducted with adjustment for confounding variables.

Multiple regression analyses were performed with different sets of covariates for each outcome. For ACE tool scores, we adjusted for sex, postgraduate year, study hours per week, enrollment in graduate school, research experience before residency, hospital characteristics (location and type), faculty academic activity (defined as the number of academic presentations by junior and senior residents of internal medicine divided by the total number of junior and senior residents of internal medicine in the hospital), and social support. GM-ITE score analyses additionally included the number of emergency department cases, weekly working hours, and number of patients in charge as covariates. For binary outcomes (depression, burnout, work satisfaction, work stress, work control, and documentation time), we further adjusted for age. Social support was assessed in nine domains using the Brief Job Stress Questionnaire, which evaluates support from superiors, co-workers, and family in terms of ease of communication, reliability in times of trouble, and willingness to listen to personal matters.

Missing data, including scholarly activity requirements, were handled using multiple imputation with 100 imputed datasets, and results were combined using Rubin’s rules. All missing values were imputed regardless of the proportion of missingness [20]. In the primary analysis of the total ACE tool score, missing responses to ACE tool items were treated as incorrect answers. Two sensitivity analyses were conducted for the primary outcome: one treating missing ACE tool responses as missing data to be imputed, and another using a mixed-effects model that included hospital as a random effect to account for clustering of residents within hospitals.

All statistical analyses were performed using R version 4.4.1, with the mice package 3.17.0 for the multiple imputation and the lme4 package 1.1.35.5 for mixed-effects modeling.

## Results

### Study population

Of 9,128 junior residents (9106 who took GM-ITE and 22 PGY-2 residents from teaching hospitals requiring scholarly activities), 1,161 residents consented to participate in the study. After excluding 9 satisficers, 1,152 residents (12.6%) were included in the analysis (Figure). Of these participants, 656 (57.0%) reported engaging in scholarly activities during their junior residency training, with 60 (9.1%) including clinical research. Baseline characteristics are shown in Table 1. Additional covariate details are shown in Supplementary Table 1.

**Table 1:** Characteristics of participants

Characteristic	Overall *N* = 1152	Without scholarly activity *N* = 496^*1*^	With scholarly activity *N* = 656	
			Without clinical research *N* = 596^*1*^	With clinical research *N* = 60^*1*^
Grade				
1	548 (48%)	310 (63%)	218 (37%)	20 (33%)
2	604 (52%)	186 (38%)	378 (63%)	40 (67%)
Women	353 (31%)	154 (31%)	188 (32%)	11 (18%)
Urban location	351 (30%)	137 (28%)	193 (32%)	21 (35%)
Hospital Type				
Community	920 (80%)	367 (74%)	502 (84%)	51 (85%)
University	165 (14%)	95 (19%)	65 (11%)	5 (8.3%)
University affiliated	67 (5.8%)	34 (6.9%)	29 (4.9%)	4 (6.7%)
Self-study time per day				
0	36 (3.1%)	16 (3.2%)	19 (3.2%)	1 (1.7%)
0–30	487 (42%)	232 (47%)	238 (40%)	17 (28%)
31–60	463 (40%)	184 (37%)	249 (42%)	30 (50%)
61–90	116 (10%)	40 (8.1%)	66 (11%)	10 (17%)
over 90	25 (2.2%)	10 (2.0%)	13 (2.2%)	2 (3.3%)
(Missing)	25 (2.2%)	14 (2.8%)	11 (1.8%)	0 (0%)
Free talk with superiors				
Extremely	182 (16%)	56 (11%)	111 (19%)	15 (25%)
Very much	376 (33%)	143 (29%)	212 (36%)	21 (35%)
Somewhat	501 (43%)	233 (47%)	250 (42%)	18 (30%)
Not at all	36 (3.1%)	17 (3.4%)	14 (2.3%)	5 (8.3%)
(Missing)	57 (4.9%)	47 (9.5%)	9 (1.5%)	1 (1.7%)
Reliability of superiors				
Extremely	251 (22%)	80 (16%)	152 (26%)	19 (32%)
Very much	460 (40%)	191 (39%)	246 (41%)	23 (38%)
Somewhat	350 (30%)	166 (33%)	170 (29%)	14 (23%)
Not at all	34 (3.0%)	14 (2.8%)	17 (2.9%)	3 (5.0%)
(Missing)	57 (4.9%)	45 (9.1%)	11 (1.8%)	1 (1.7%)
Support from superiors				
Extremely	200 (17%)	66 (13%)	117 (20%)	17 (28%)
Very much	397 (34%)	162 (33%)	214 (36%)	21 (35%)
Somewhat	437 (38%)	198 (40%)	226 (38%)	13 (22%)
Not at all	57 (4.9%)	24 (4.8%)	26 (4.4%)	7 (12%)
(Missing)	61 (5.3%)	46 (9.3%)	13 (2.2%)	2 (3.3%)
Graduate school	43 (3.7%)	8 (1.6%)	30 (5.0%)	5 (8.3%)
Scholarly activity before junior residency	244 (21%)	51 (10%)	171 (29%)	22 (37%)
Requirement of scholarly activity in the program				
No	195 (17%)	84 (17%)	97 (16%)	14 (23%)
Yes	143 (12%)	56 (11%)	72 (12%)	15 (25%)
(Missing)	814 (71%)	356 (72%)	427 (72%)	31 (52%)
JSIM presentations per resident				
Median (Q1, Q3)	0.33 (0.21, 0.59)	0.33 (0.22, 0.54)	0.34 (0.20, 0.62)	0.30 (0.17, 0.62)
(Missing)	230 (20%)	108 (22%)	110 (18%)	12 (20%)

*Abbreviation*: *JSIM* the Japanese Society of Internal Medicine

^*1*^n (%)

### Characteristics of scholarly activities

Case reports were the predominant type of scholarly activity, while conference presentations were the most common format. Detailed distributions of scholarly activity types, presentation formats, and language use are presented in Table 2.

**Table 2:** Types and characteristics of scholarly activities during residency

Characteristic	Overall *N* = 656^*1*^	Not required *N* = 111^*1*^	Required *N* = 87^*1*^	Unknown *N* = 458^*1*^
Type of scholarly activity				
Case report	558 (85%)	90 (81%)	68 (78%)	400 (87%)
Clinical research	60 (9.1%)	14 (13%)	15 (17%)	31 (6.8%)
Medical education	22 (3.4%)	3 (2.7%)	4 (4.6%)	15 (3.3%)
Quality improvement	10 (1.5%)	1 (0.9%)	0 (0%)	9 (2.0%)
Basic research	33 (5.0%)	7 (6.3%)	6 (6.9%)	20 (4.4%)
Letter to editor	11 (1.7%)	1 (0.9%)	4 (4.6%)	6 (1.3%)
Format of presentation				
In-house presentation	323 (49%)	43 (39%)	46 (53%)	234 (51%)
Conference presentation	445 (68%)	72 (65%)	55 (63%)	318 (69%)
Paper publication	59 (9.0%)	12 (11%)	10 (11%)	37 (8.1%)
Other presentation	48 (7.3%)	11 (9.9%)	8 (9.2%)	29 (6.3%)
Language				
Japanese	610 (95%)	100 (93%)	83 (97%)	427 (96%)
English	61 (9.5%)	12 (11%)	14 (16%)	35 (7.8%)
Other language	7 (1.1%)	2 (1.9%)	0 (0%)	5 (1.1%)

^*1*^n (%)

### Primary outcome: EBM competency

After adjusting for potential confounders and confirming the convergence of variables in multiple imputation (Supplementary figure), the adjusted difference in ACE tool scores between residents with and without scholarly activity (reference group) was 0.22 points (95% confidence interval [CI]: 0.02 to 0.41) for residents who engaged in scholarly activities without clinical research and 0.40 points (95% CI: -0.02 to 0.83) for those who conducted clinical research (Table 3, Supplementary table 2a). When analyzing by program requirements, the adjusted difference in ACE tool scores was 0.34 points (95% CI: 0.09 to 0.58) for residents who engaged in scholarly activities without requirements and 0.09 points (95% CI: -0.19 to 0.37) for those with requirements, compared to residents without scholarly activity (Table 4, Supplementary table 2). Similar patterns were observed in sensitivity analyses using multiple imputation for missing ACE tool responses, when accounting for hospital-level clustering, and participants divided by the presence of the mentor in the program (Table 5, Supplementary Table 3 ).

**Table 3:** Association between clinical research involvement and outcomes among residents

	Total ACE tool score	Total GM- ITE score	Future academic interest	Depression	Burnout
	β (95% CI)	β (95% CI)	OR (95% CI)	OR (95% CI)	OR (95% CI)
No scholarly activity	Reference	Reference	Reference	Reference	Reference
Scholarly activity without clinical research*	0.22 (0.02, 0.41)	0.67 (-0.21, 1.55)	1.27 (0.97, 1.68)	1.64 (1.13, 2.38)	1.23 (0.88, 1.73)
Scholarly activity with clinical research	0.40 (-0.02, 0.83)	-1.05 (-3, 0.9)	2.68 (1.33, 5.37)	1.85 (0.86, 3.99)	1.32 (0.65, 2.68)

All analyses were adjusted for potential confounders. See main text for details of covariates used in each analysis

*Abbreviations*: *ACE* Assessing Competency for Evidence-Based Medicine, *GM-ITE* General Medicine In-Training Examination

*Scholarly activity defined as case reports, quality improvement studies, and/or medical education research

**Table 4:** Association between required scholarly activity and outcomes among residents

	Total ACE tool score	Total GM ITE score	Future academic interest	Depression	Burnout
	β (95% CI)	β (95% CI)	OR (95% CI)	OR (95% CI)	OR (95% CI)
No scholarly activity	Reference	Reference	Reference	Reference	Reference
Scholarly activity without requirement	0.34 (0.09, 0.58)	0.59 (-0.56, 1.74)	1.24 (0.88, 1.74)	1.68 (1.05, 2.7)	1.42 (0.94, 2.13)
Scholarly activity with requirement	0.09 (-0.19, 0.37)	0.43 (-0.88, 1.74)	1.52 (1.02, 2.25)	1.6 (0.91, 2.8)	1.02 (0.63, 1.66)

All analyses were adjusted for potential confounders. See main text for details of covariates used in each analysis

*Abbreviations*: *ACE* Assessing Competency for Evidence-Based Medicine, *GM-ITE* General Medicine In-Training Examination

**Table 5:** Association between the presence of mentor in the program and outcomes among Japanese residents

	Total ACE tool score	Total GM ITE score	Future academic interest	Depression	Burnout	Work satisfaction	Work stress	Work control	Documentation time during work hours	Time spent on documentation after work
	β (95% CI)	β (95% CI)	OR (95% CI)	OR (95% CI)	OR (95% CI)	OR (95% CI)	OR (95% CI)	OR (95% CI)	OR (95% CI)	OR (95% CI)
No scholarly activity (*N* = 496)	Reference	Reference	Reference	Reference	Reference	Reference	Reference	Reference	Reference	Reference
Scholarly activity without mentor in the program	0.13 (-0.36, 0.63)	0.17 (-2.15, 2.5)	1.21 (0.6, 2.46)	1.43 (0.6, 3.42)	2.56 (1.23, 5.33)	0.73 (0.34, 1.58)	1.1 (0.54, 2.23)	0.61 (0.25, 1.49)	0.53 (0.23, 1.2)	1.42 (0.63, 3.18)
Scholarly activity with mentor in the program	0.16 (-0.05, 0.37)	0.58 (-0.36, 1.51)	1.34 (1.00, 1.80)	1.69 (1.14, 2.5)	1.06 (0.73, 1.54)	1.16 (0.83, 1.62)	1.11 (0.84, 1.49)	1.1 (0.69, 1.76)	1.15 (0.72, 1.83)	0.9 (0.60, 1.34)

All analyses were adjusted for potential confounders. See Supplementary methods section for details of covariates used in each analysis

*Abbreviations*: *ACE* Assessing Competency for Evidence-Based Medicine, *GM-ITE* General Medicine In-Training Examination; EMR, electric medical records

### Secondary outcome: GM-ITE Score

In the analysis by clinical research experience, GM-ITE scores were similar between residents who engaged in scholarly activities (with or without clinical research) and the reference group (adjusted differences: 0.67 points [95% CI: -0.21 to 1.55] and -1.05 points [95% CI: -3.00 to 0.90], respectively) (Table 3). Similarly, in the analysis by program requirements, GM-ITE scores were similar across all groups (adjusted differences: 0.59 points [95% CI: -0.56 to 1.74] for those without requirements and 0.43 points [95% CI: -0.88 to 1.74] for those with requirements) (Table 4).

### Secondary outcome: future academic interest

In the analysis by clinical research experience, future academic interest was similar between residents who engaged in scholarly activities without clinical research and the reference group (OR: 1.27 [95% CI: 0.97 to 1.68]), while residents who conducted clinical research showed higher odds of future academic interest (OR: 2.68 [95% CI: 1.33 to 5.37]) (Table 3).

In the analysis by program requirements, future academic interest was similar among residents without requirements (OR: 1.24 [95% CI: 0.88 to 1.74]), while residents with requirements showed higher odds of future academic interest (OR: 1.52 [95% CI: 1.02 to 2.25]), compared to the reference group (Table 4). 

### Secondary outcome: depression and well-being

In the analysis by clinical research experience, the odds ratios for depression symptoms were 1.64 (95% CI: 1.13 to 2.38) for residents who engaged in scholarly activities without clinical research and 1.85 (95% CI: 0.86 to 3.99) for those who conducted clinical research, compared to those without scholarly activity. For the Mini-Z items (burnout, work satisfaction, work stress, work control, and time spent on documentation), the odds ratios comparing each group to residents without scholarly activity were similar (Table 3).

In the analysis by program requirements, the odds ratios for depression symptoms were 1.68 (95% CI: 1.05 to 2.70) for residents who engaged in scholarly activities without requirements and 1.60 (95% CI: 0.91 to 2.80) for those with requirements, compared to those without scholarly activity. For the Mini-Z items, the odds ratios comparing each group to residents without scholarly activity were similar (Table 4).

## Discussion

In this cross-sectional study of 1,152 junior residents, including 656 residents with scholarly activity experience and 60 with clinical research experience, we examined the impact of scholarly activities from two perspectives: the presence of clinical research experience and the requirement of scholarly activities by programs. The adjusted difference in ACE tool scores between residents with and without scholarly activity was small, ranging from 0.22 to 0.40 points for those with clinical research experience and from 0.09 to 0.34 points when analyzed by program requirements. Residents who engaged in scholarly activities with clinical research showed increased interest in future academic activities compared to those without scholarly activities. Associations with depression symptoms were heterogeneous across subgroups. Higher odds of PHQ-2 positivity were observed among residents engaged in scholarly activities without clinical research and among those participating without program requirements, whereas odds were similar to the reference group among those engaged in scholarly activities with clinical research or among those in programs with requirements. Other outcomes including burnout, work satisfaction, work stress, and work control were comparable between groups, whether compared by clinical research experience or by program requirements. Overall, the presence or absence of a scholarly activities requirement in residency programs did not demonstrate clear advantages or disadvantages in terms of residents' competency development or well-being measures.

While previous studies have suggested educational benefits of scholarly activities during mid postgraduate training (PGY3-6), our findings indicate more nuanced effects during early postgraduate training (PGY1-2). Although scholarly activities during mid postgraduate training are generally considered to enhance residents' EBM competency and clinical performance, this assumption is largely based on expert opinions and single-center studies of senior residents, with some research suggesting no significant relationship between scholarly activities and clinical performance [9, 10, 21, 22]. The contrasting findings between our study of early postgraduate trainees and previous studies of mid-career residents may reflect fundamental differences between early clinical training years, when trainees are primarily focused on acquiring basic clinical skills, and mid-career years when they may be better equipped to integrate research and clinical practice. Our study provides empirical evidence that questions the assumed benefits of scholarly activities during early training years, suggesting that timing of research exposure may be a critical factor to consider.

Our findings suggest that scholarly activities requirements for all junior residents may not be necessary, but supporting those who show interest in clinical research could be valuable for developing future physician-scientists. We found that scholarly activities with clinical research were not associated with decreased clinical competency or substantial deterioration in well-being measures, suggesting that providing clinical research opportunities for interested residents is a reasonable approach. The observation that residents who engaged in scholarly activities with clinical research showed increased interest in future academic activities, aligns with previous studies suggesting early research experience as a predictor of future academic engagement [22, 23]. This consistent finding across studies highlights the potential role of scholarly activities with clinical research during junior residency as a pathway for identifying and nurturing future physician-scientists. Regarding the timing of such opportunities, programs with shorter training durations may feel pressure to initiate scholarly activities as early as PGY1; however, our study does not identify an optimal start point or support a need for universal early participation. Rather than prescribing a specific start point, programs should foster supportive environments in which trainees who wish to pursue scholarly activities—including those in PGY1—are encouraged to do so. The challenge lies in developing appropriate support systems that can facilitate meaningful research experiences while ensuring residents' well-being and clinical training quality.

Based on our study findings, we propose two levels of systematic reforms to provide effective research support during residency. First, residency programs should offer a structured research block option that allows interested residents to conduct research during their PGY-2 year. While the effectiveness of protected time for research is well-established in postgraduate medical education23, many clinical educators in Japan may not fully appreciate its importance, as evidenced by previous survey among internal medicine residency program directors showing that only 22-32% of teaching hospitals provide protected research time [24]. Program directors need to understand that early research experience during residency can foster long-term academic engagement and career development.

Second, reforms at the national level are needed to address the shortage of research mentors. Even when programs attempt to recruit external mentors, many potential advisors are overwhelmed with clinical duties, limiting their availability for mentorship. This reflects broader systemic issues in Japanese healthcare, where physicians often handle tasks that could be delegated to other professionals. Progress in task-shifting to nurses and pharmacists, along with digital transformation of healthcare administrative work, could reduce physicians' non-clinical workload. Such changes would create more capacity for experienced clinicians to serve as research mentors. However, the effectiveness of these reforms in supporting residents' scholarly activities and their impact on long-term career development remains to be evaluated through rigorous research. As a constructive plan, one possible approach could be to further clarify the roles of institutions in Japan, such as university hospitals and community-based hospitals, while steering the direction of training at academic centers toward a more research-oriented focus. This strategy might serve as a means to prevent the dispersion of the already limited number of research mentors.

Several limitations should be considered when interpreting our findings. First, although the Japanese version of the ACE tool demonstrated acceptable validity, its relatively low internal consistency (Cronbach alpha = 0.31) suggests that the tool may not fully capture EBM competency, potentially measuring other constructs as well. Second, our study only assessed competency during residency training (at the end of first year or second year); the long-term impact of scholarly activities on residents' clinical practice, EBM competency, and patient outcomes remains unknown. Third, there may be selection bias in our study population due to the low response rate and voluntary participation. Of 9,106 eligible residents from GM-ITE taker, 1,159 (12.7%) provided consent, and an additional 9 were excluded as satisficers, resulting in a final sample of 1,150 (our total sample of 1,152 includes the remaining 2 residents from non-participating hospitals). Participants who volunteered may differ systematically from non-participants, and the very low participation from non-GM-ITE residents (2 out of 22 eligible) further limits the representativeness of our sample, potentially limiting the generalizability of our findings. Despite these limitations, the relatively large sample size of 1,152 residents from multiple institutions strengthens the reliability of our findings and provides valuable insights into the immediate impact of scholarly activities during junior residency training. 

## Conclusion

In this cross-sectional study of Japanese junior residents,scholarly activities during residency training had little impact on EBM competency or well-being, regardless of whether they were program-required. However, residents who engaged in clinical research showed increased interest in future academic activities, suggesting potential value in fostering research experiences for interested trainees rather than implementing universal requirements. Future studies should evaluate the long-term impact of early research experience on physicians' career development.

## Supplementary Information

Supplementary Material 1.

## Data Availability

The data that support the findings of this study are available from the Japan Institute for Advancement of Medical Education Program (JAMEP) but restrictions apply to the availability of these data, which were used under license for the current study, and so are not publicly available. Data are however available from the authors upon reasonable request and with permission of JAMEP.
