# Supplementary material for: Impact of scholarly activities on early postgraduate medical trainees: a cross-sectional study
Source: BMC Med Educ. 2026 May 1;26:998. doi: 10.1186/s12909-026-09272-x (PMC13281296; doi:10.1186/s12909-026-09272-x)

# Supplementary table 1 Survey questionnaires

| Question | Response format | Choices |
| --- | --- | --- |
| Age | Single choice | (1) 24 years (2) 25 years (3) 26 years (4) 27 years (5) 28 years (6) 29 years (7) 30 years or older |
| Sex | Single choice | (1) Male (2) Female (3) Prefer not to answer |
| Post Graduate Year (PGY) | Single choice | (1) PGY-1 (2) PGY-2 |
| Desired specialty | Multiple choice | (1) Internal Medicine (2) Surgery (3) Pediatrics (4) Obstetrics and Gynecology (5) Psychiatry (6) Dermatology (7) Ophthalmology (8) Otolaryngology (9) Urology (10) Orthopedic Surgery (11) Neurosurgery (12) Plastic Surgery (13) Emergency Medicine (14) Anesthesiology (15) Radiology (16) Rehabilitation Medicine (17) Pathology (18) Clinical Laboratory (19) General Medicine (20) Other (21) Undecided |
| Average number of ER night shifts per month | Single choice | (1) 0 (2) 1–2 (3) 3–5 (4) 6 or more (5) Unknown |
| Average number of inpatients assigned at any given time | Single choice | (1) 0–4 (2) 5–9 (3) 10–14 (4) 15 or more (5) Unknown |
| Average daily self-study time (desk study) | Single choice | (1) 0–30 min (2) 31–60 min (3) 61–90 min (4) 91 min or more (5) None |
| Average working hours per week (including on-call standby time) | Single choice | (1) Less than 45 hours (2) 45 to <50 hours (3) 50 to <55 hours (4) 55 to <60 hours (5) 60 to <65 hours (6) 65 to <70 hours (7) 70 to <80 hours (8) 80 to <90 hours (9) 90 to <100 hours (10) 100 hours or more |
| Have you experienced scholarly activities during your junior residency? A scholarly activity is defined as presenting a case report (including case series), basic research, clinical research, medical education research, or quality of care assessment as the first author in any form, such as a conference presentation or paper, regardless of the language (e.g., Japanese or English) or format (e.g., poster or slide presentation). | Single choice | (1) Yes (2) No |
| (For those who answered “Yes” to the previous question) What types of scholarly activities have you experienced? (Select all that apply) | Multiple choice | (1) Case report (including case series or clinical images) (2) Clinical research (3) Medical education research (4) Healthcare quality assessment (5) Letter to the editor (6) Basic research (7) I have not experienced scholarly activities |
| (For those who answered “Yes” to the previous question) What formats did you use to present your scholarly activities? (Select all that apply) | Multiple choice | (1) In-hospital presentation (e.g., case conferences or research presentations mimicking academic meetings, excluding CPCs or dry runs) (2) Presentation at an academic conference (annual or regional meeting) (3) Presentation at study groups hosted by non-academic organizations (e.g., pharmaceutical companies) (4) Publication of a paper (including submitted but not yet accepted works) (5) I have not experienced scholarly activities |
| (For those who answered “Yes” to the previous question) In what language(s) did you conduct your scholarly activities? (Select all that apply) | Multiple choice | (1) Japanese (2) English (3) Other |
| Are you currently enrolled in a graduate school (including schools of public health) as of January 2024? | Single choice | (1) Yes (2) No |
| Did you have any scholarly activity experience prior to starting your junior residency? (including first-author or co-author roles) | Single choice | (1) Yes (2) No |
| Over the past month, have you often been bothered by feeling down, depressed, or hopeless? | Single choice | (1) Yes (2) No |
| Over the past month, have you often been bothered by having little interest or pleasure in doing things? | Single choice | (1) Yes (2) No |
| Using your own definition of “burnout”, please select the statement that best describes your situation: | Single choice | (1) I feel completely burned out and may need to seek some help (2) The symptoms of burnout that I’m experiencing won’t go away. I think about frustration at work a lot (3) I am definitely burning out and have one or more symptoms of burnout, such as physical and emotional exhaustion (4) I am under stress, and don’t always have as much energy as I once did, but I don’t feel burned out (5) I enjoy my work. I have no symptoms of burnout |
| Overall, I am satisfied with my current job. | Single choice | (1) Strongly agree (2) Agree (3) Neither agree nor disagree (4) Disagree (5) Strongly disagree |
| I feel a great deal of stress because of my job. | Single choice | (1) Strongly agree (2) Agree (3) Neither agree nor disagree (4) Disagree (5) Strongly disagree |
| My control over my workload is: | Single choice | (1) Optimal (2) Good (3) Satisfactory (4) Marginal (5) Poor |
| Sufficiency of time for documentation is: | Single choice | (1) Optimal (2) Good (3) Satisfactory (4) Marginal (5) Poor |
| The amount of time spent on the electronic medical record (EMR) at home is: | Single choice | (1) Minimal/None (2) Modest (3) Moderate (4) High (5) Excessive |
| How freely can you talk with the following people? - Supervisors | Single choice | (1) Very much (2) Considerably (3) Somewhat (4) Not at all |
| How freely can you talk with the following people? - Co-workers | Single choice | (1) Very much (2) Considerably (3) Somewhat (4) Not at all |
| How freely can you talk with the following people? - Spouse, family, friends, etc. | Single choice | (1) Very much (2) Considerably (3) Somewhat (4) Not at all |
| How reliable are the following people when you are troubled? - Supervisors | Single choice | (1) Very much (2) Considerably (3) Somewhat (4) Not at all |
| How reliable are the following people when you are troubled? - Co-workers | Single choice | (1) Very much (2) Considerably (3) Somewhat (4) Not at all |
| How reliable are the following people when you are troubled? - Spouse, family, friends, etc. | Single choice | (1) Very much (2) Considerably (3) Somewhat (4) Not at all |
| How well will the following people listen to you when you consult them about personal problems? - Supervisors | Single choice | (1) Very much (2) Considerably (3) Somewhat (4) Not at all |
| How well will the following people listen to you when you consult them about personal problems? - Co-workers | Single choice | (1) Very much (2) Considerably (3) Somewhat (4) Not at all |
| How well will the following people listen to you when you consult them about personal problems? - Spouse, family, friends, etc. | Single choice | (1) Very much (2) Considerably (3) Somewhat (4) Not at all |
| Do you want to engage in scholarly activities in the future? | Single choice | (1) Strongly agree (2) Agree (3) Neither agree nor disagree (4) Disagree (5) Strongly disagree |

| Supplementary table 2 Covariates that were used in the analyses of secondary outcomes such as depression | | | | |
| --- | --- | --- | --- | --- |
| **Characteristic** | **Overall**  N = 1,152*^1^* | **Without scholarly activity**  N = 496*^1^* | **With scholarly activity N = 656** | |
|  |  |  | **Without clinical research** N = 596*^1^* | **With clinical research** N = 60*^1^* |
| Age |  |  |  |  |
| 24 | 37 (3.2%) | 19 (3.8%) | 17 (2.9%) | 1 (1.7%) |
| 25 | 223 (19%) | 119 (24%) | 97 (16%) | 7 (12%) |
| 26 | 333 (29%) | 138 (28%) | 181 (30%) | 14 (23%) |
| 27 | 236 (20%) | 85 (17%) | 133 (22%) | 18 (30%) |
| 28 | 95 (8.2%) | 36 (7.3%) | 52 (8.7%) | 7 (12%) |
| 29 | 64 (5.6%) | 28 (5.6%) | 33 (5.5%) | 3 (5.0%) |
| over 30 | 155 (13%) | 63 (13%) | 82 (14%) | 10 (17%) |
| (Missing) | 9 (0.8%) | 8 (1.6%) | 1 (0.2%) | 0 (0%) |
| ED duties per month |  |  |  |  |
| 0 | 45 (3.9%) | 23 (4.6%) | 21 (3.5%) | 1 (1.7%) |
| 1-2 | 213 (18%) | 97 (20%) | 110 (18%) | 6 (10%) |
| 3-5 | 784 (68%) | 336 (68%) | 402 (67%) | 46 (77%) |
| over 6 | 95 (8.2%) | 31 (6.3%) | 57 (9.6%) | 7 (12%) |
| (Missing) | 15 (1.3%) | 9 (1.8%) | 6 (1.0%) | 0 (0%) |
| Assigned inpatients |  |  |  |  |
| 0-4 | 409 (36%) | 192 (39%) | 201 (34%) | 16 (27%) |
| 10-14 | 107 (9.3%) | 32 (6.5%) | 66 (11%) | 9 (15%) |
| 5-9 | 575 (50%) | 241 (49%) | 305 (51%) | 29 (48%) |
| over 15 | 31 (2.7%) | 10 (2.0%) | 16 (2.7%) | 5 (8.3%) |
| (Missing) | 30 (2.6%) | 21 (4.2%) | 8 (1.3%) | 1 (1.7%) |
| Weekly duty hours |  |  |  |  |
| under 45 | 62 (5.4%) | 33 (6.7%) | 28 (4.7%) | 1 (1.7%) |
| 45-50 | 193 (17%) | 87 (18%) | 97 (16%) | 9 (15%) |
| 50-55 | 222 (19%) | 87 (18%) | 123 (21%) | 12 (20%) |
| 55-60 | 160 (14%) | 76 (15%) | 74 (12%) | 10 (17%) |
| 60-65 | 131 (11%) | 64 (13%) | 62 (10%) | 5 (8.3%) |
| 65-70 | 98 (8.5%) | 45 (9.1%) | 47 (7.9%) | 6 (10%) |
| 70-80 | 104 (9.0%) | 28 (5.6%) | 70 (12%) | 6 (10%) |
| 80-85 | 1 (<0.1%) | 0 (0%) | 0 (0%) | 1 (1.7%) |
| 80-90 | 100 (8.7%) | 40 (8.1%) | 52 (8.7%) | 8 (13%) |
| 90-100 | 36 (3.1%) | 14 (2.8%) | 21 (3.5%) | 1 (1.7%) |
| over 100 | 24 (2.1%) | 10 (2.0%) | 13 (2.2%) | 1 (1.7%) |
| (Missing) | 21 (1.8%) | 12 (2.4%) | 9 (1.5%) | 0 (0%) |
| Free talk with coworkers |  |  |  |  |
| Extremely | 428 (37%) | 161 (32%) | 241 (40%) | 26 (43%) |
| Very much | 447 (39%) | 190 (38%) | 233 (39%) | 24 (40%) |
| Somewhat | 201 (17%) | 95 (19%) | 100 (17%) | 6 (10%) |
| Not at all | 16 (1.4%) | 4 (0.8%) | 9 (1.5%) | 3 (5.0%) |
| (Missing) | 60 (5.2%) | 46 (9.3%) | 13 (2.2%) | 1 (1.7%) |
| Free talk with family/friends |  |  |  |  |
| Extremely | 535 (46%) | 219 (44%) | 283 (47%) | 33 (55%) |
| Very much | 406 (35%) | 174 (35%) | 214 (36%) | 18 (30%) |
| Somewhat | 139 (12%) | 54 (11%) | 80 (13%) | 5 (8.3%) |
| Not at all | 16 (1.4%) | 4 (0.8%) | 9 (1.5%) | 3 (5.0%) |
| (Missing) | 56 (4.9%) | 45 (9.1%) | 10 (1.7%) | 1 (1.7%) |
| Reliability of coworkers |  |  |  |  |
| Extremely | 309 (27%) | 105 (21%) | 185 (31%) | 19 (32%) |
| Very much | 475 (41%) | 202 (41%) | 249 (42%) | 24 (40%) |
| Somewhat | 284 (25%) | 133 (27%) | 139 (23%) | 12 (20%) |
| Not at all | 29 (2.5%) | 12 (2.4%) | 13 (2.2%) | 4 (6.7%) |
| (Missing) | 55 (4.8%) | 44 (8.9%) | 10 (1.7%) | 1 (1.7%) |
| Reliability of family/friends |  |  |  |  |
| Extremely | 447 (39%) | 176 (35%) | 245 (41%) | 26 (43%) |
| Very much | 443 (38%) | 198 (40%) | 226 (38%) | 19 (32%) |
| Somewhat | 192 (17%) | 72 (15%) | 108 (18%) | 12 (20%) |
| Not at all | 13 (1.1%) | 5 (1.0%) | 7 (1.2%) | 1 (1.7%) |
| (Missing) | 57 (4.9%) | 45 (9.1%) | 10 (1.7%) | 2 (3.3%) |
| Support from coworkers |  |  |  |  |
| Extremely | 297 (26%) | 104 (21%) | 174 (29%) | 19 (32%) |
| Very much | 474 (41%) | 206 (42%) | 243 (41%) | 25 (42%) |
| Somewhat | 296 (26%) | 129 (26%) | 155 (26%) | 12 (20%) |
| Not at all | 25 (2.2%) | 8 (1.6%) | 14 (2.3%) | 3 (5.0%) |
| (Missing) | 60 (5.2%) | 49 (9.9%) | 10 (1.7%) | 1 (1.7%) |
| Support from family/friends |  |  |  |  |
| Extremely | 479 (42%) | 193 (39%) | 259 (43%) | 27 (45%) |
| Very much | 439 (38%) | 191 (39%) | 228 (38%) | 20 (33%) |
| Somewhat | 164 (14%) | 61 (12%) | 93 (16%) | 10 (17%) |
| Not at all | 8 (0.7%) | 3 (0.6%) | 4 (0.7%) | 1 (1.7%) |
| (Missing) | 62 (5.4%) | 48 (9.7%) | 12 (2.0%) | 2 (3.3%) |
| *^1^*n (%) | | | | |

# Supplementary table 3 Additional well-being outcomes among residents

1. Scholarly activity with/without clinical research

|  | **Work satisfaction** | **Work stress** | **Work control** | **Documentation time during work hours** | **Time spent on documentation after work** |
| --- | --- | --- | --- | --- | --- |
|  | OR (95% CI) | OR (95% CI) | OR (95% CI) | OR (95% CI) | OR (95% CI) |
| No scholarly activity | Reference | Reference | Reference | Reference | Reference |
| Scholarly activity without clinical research* | 1.09 (0.8, 1.49) | 1.13 (0.86, 1.48) | 1.03 (0.67, 1.59) | 1.04 (0.67, 1.6) | 0.95 (0.66, 1.38) |
| Scholarly activity with clinical research | 1.16 (0.57, 2.39) | 1.07 (0.59, 1.95) | 0.65 (0.28, 1.5) | 0.6 (0.27, 1.34) | 1.25 (0.59, 2.64) |

*Scholarly activity defined as case reports, quality improvement studies, and/or medical education research

Note: All analyses were adjusted for potential confounders. See Supplementary methods section for details of covariates used in each analysis.

1. Scholarly activity with/without requirements

|  | **Work satisfaction** | **Work stress** | **Work control** | **Documentation time during work hours** | **Time spent on documentation after work** |
| --- | --- | --- | --- | --- | --- |
|  | OR (95% CI) | OR (95% CI) | OR (95% CI) | OR (95% CI) | OR (95% CI) |
| No scholarly activity | Reference | Reference | Reference | Reference | Reference |
| Scholarly activity without requirement | 1.1 (0.73, 1.65) | 1.32 (0.93, 1.88) | 0.87 (0.51, 1.46) | 0.83 (0.5, 1.38) | 1.22 (0.79, 1.9) |
| Scholarly activity with requirement | 1.09 (0.68, 1.75) | 0.9 (0.59, 1.35) | 1.21 (0.64, 2.29) | 1.25 (0.68, 2.29) | 0.69 (0.4, 1.21) |

Note: All analyses were adjusted for potential confounders. See Supplementary methods

# Supplementary table 4 Results of sensitivity analysis

1. Multiple imputation for the answers of ACE tool classified by with/without clinical research

|  | **Total ACE tool score** |
| --- | --- |
|  | β (95% CI) |
| No scholarly activity  (N= 496 ) | Reference |
| Scholarly activity without clinical research*  (N= 596) | 0.15 (-0.05, 0.35) |
| Scholarly activity with clinical research  (N= 60) | 0.18 (-0.23, 0.59) |

*Scholarly activity defined as case reports, quality improvement studies, and/or medical education research

1. Multiple imputation for the answers of ACE tool classified by with/without requirement of scholarly activity

|  | **Total ACE tool score** |
| --- | --- |
|  | β (95% CI) |
| No scholarly activity  (N= 496) | Reference |
| Scholarly activity without requirement | 0.26 (0, 0.51) |
| Scholarly activity with requirement | 0.01 (-0.26, 0.29) |

1. Fixed effects estimates and 95% confidence intervals from multilevel linear regression for total ACE score by clinical research experience, accounting for hospital-level random effects

|  | **Total ACE tool score** |
| --- | --- |
|  | β (95% CI) |
| No scholarly activity  (N= 496 ) | Reference |
| Scholarly activity without clinical research*  (N= 596) | 0.22 (0.02, 0.42) |
| Scholarly activity with clinical research  (N= 60) | 0.41 (-0.01, 0.84) |

1. Fixed effects estimates and 95% confidence intervals from multilevel linear regression for total ACE score by scholarly activity requirement, accounting for hospital-level random effects

|  | **Total ACE tool score** |
| --- | --- |
|  | β (95% CI) |
| No scholarly activity  (N= 496) | Reference |
| Scholarly activity without requirement | 0.34 (0.09, 0.59) |
| Scholarly activity with requirement | 0.10 (-0.39, 0.38) |

# Supplementary Figure Convergence plots for multiply imputed variables

1. Age
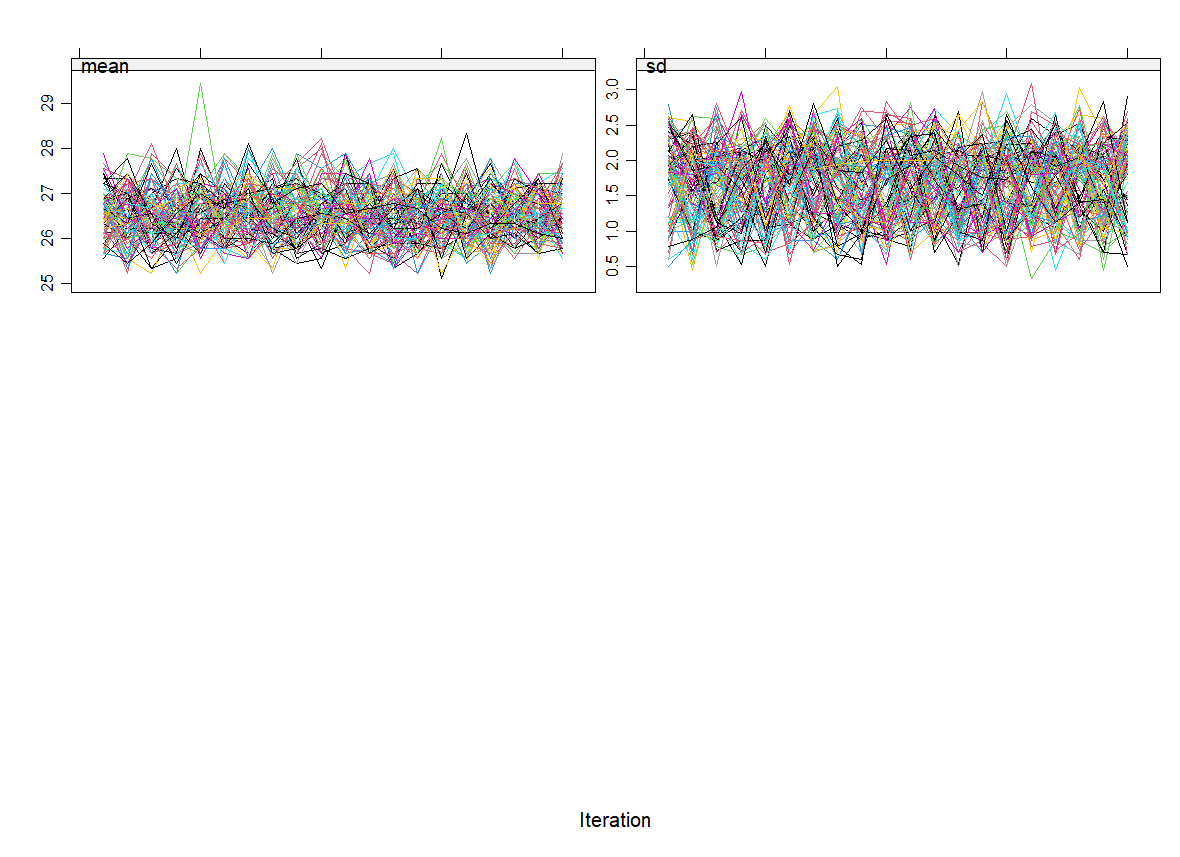

2. Number of ER duties
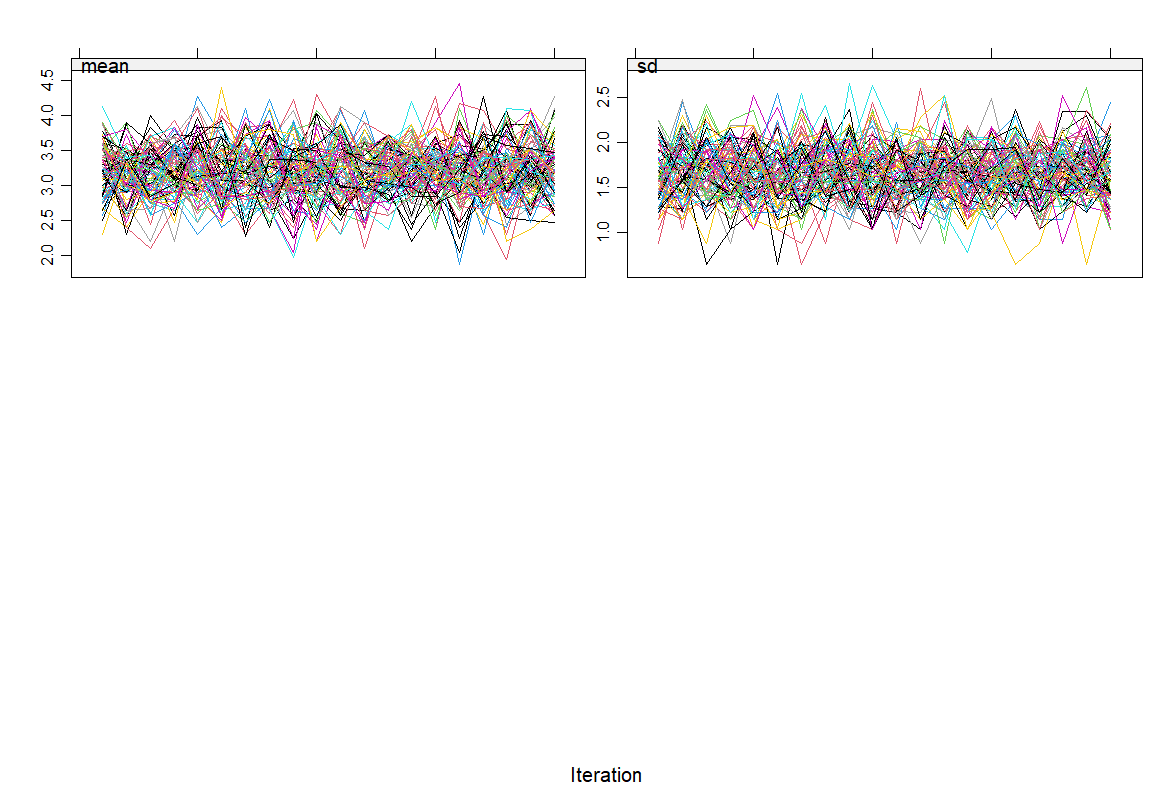

3. Number of patient incharge
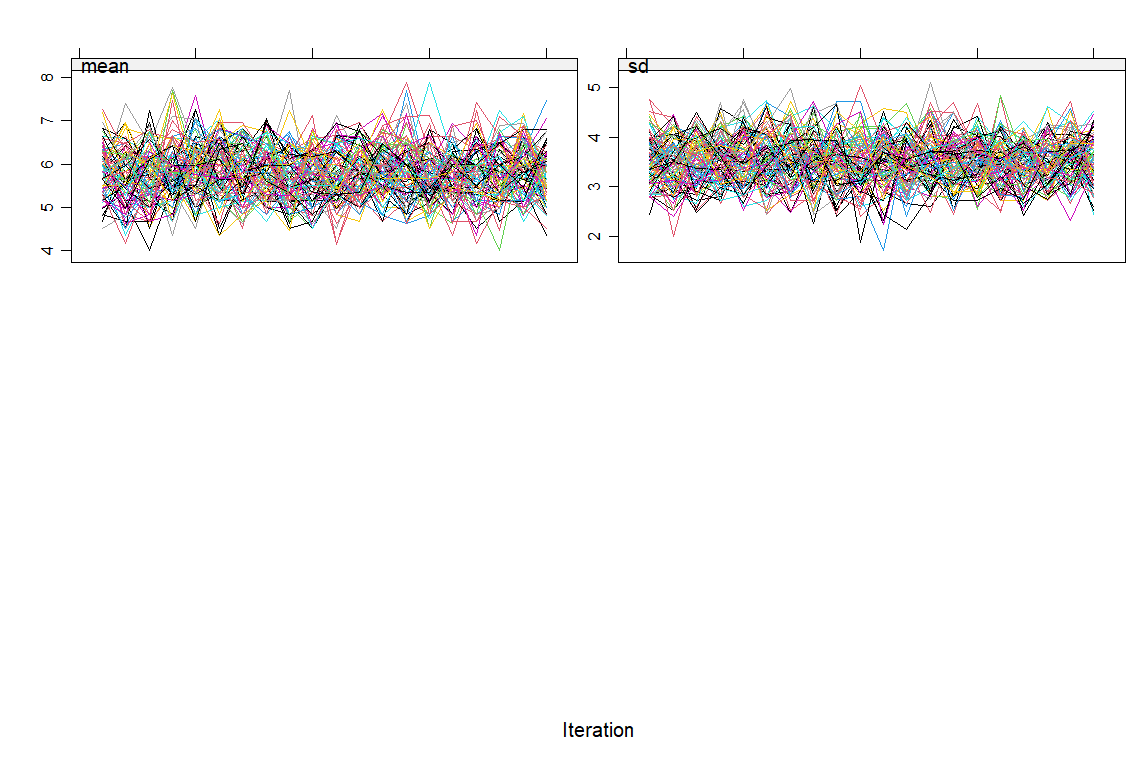

4. Self study hours
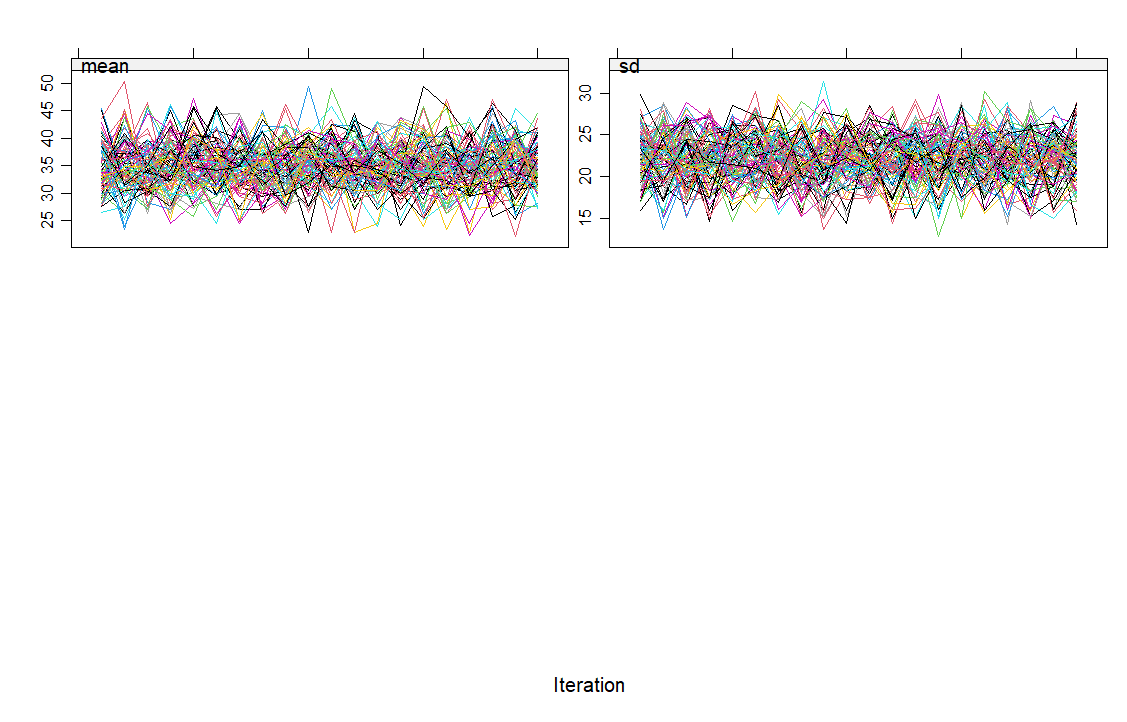

5. Working hours
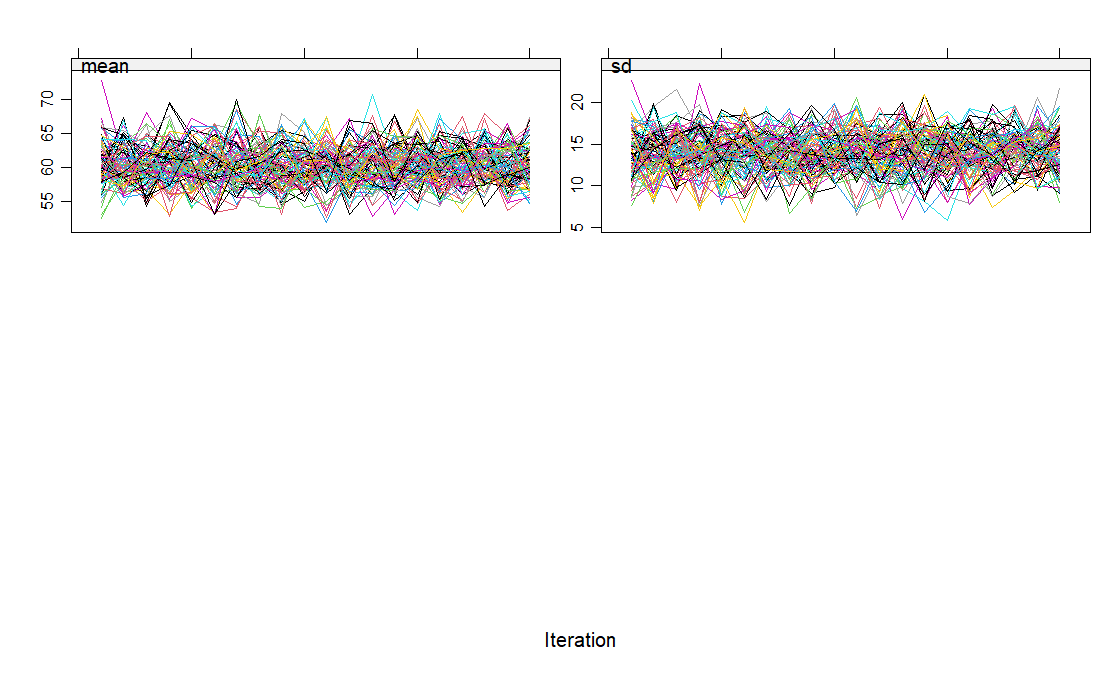

6. Requirement of scholarly activity
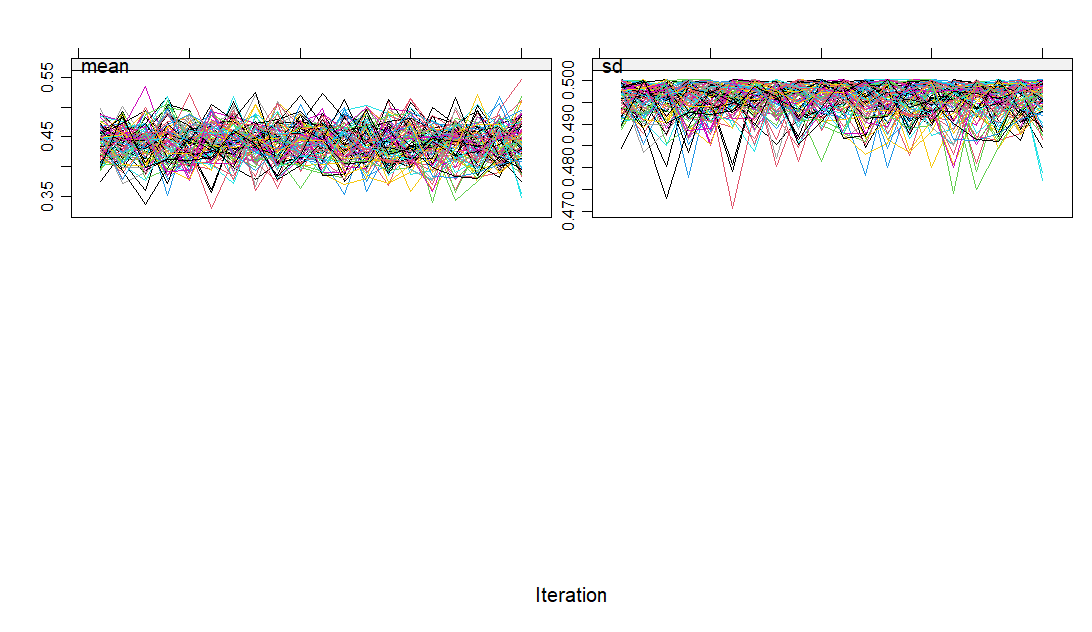

7. Mentor in the program


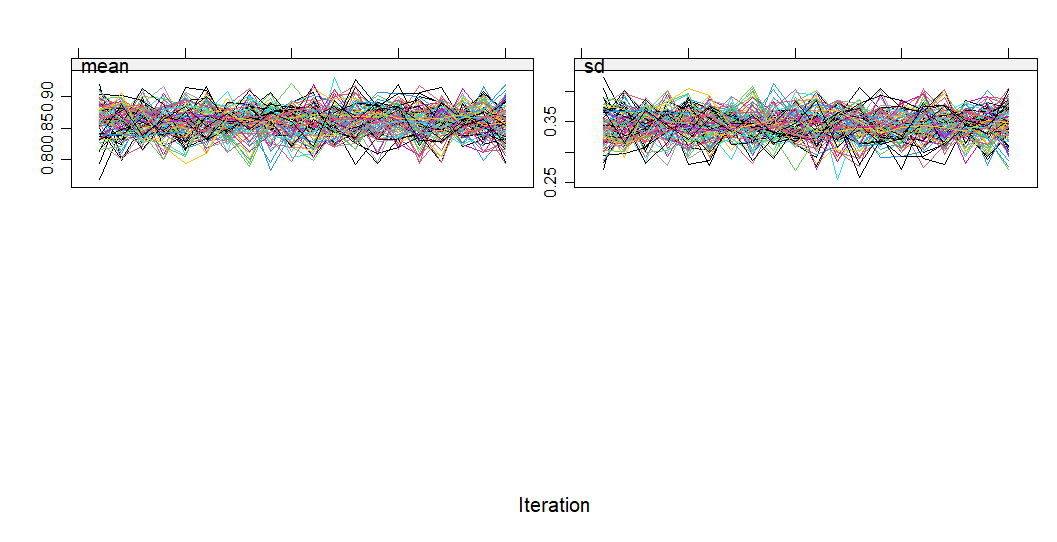

Supplement: Supplementary file 1 — Supplementary Material 1. [file 12909_2026_9272_MOESM1_ESM.docx]
